# 

**Published:** 1912-08-15

**Authors:** 


### ANNOUNCEMENTS.

NATIONAL DENTAL ASSOCIATION. The 1912 session of the National Dental Association will be held in Washington, D. C., September 10th to 13th, and all indications are favorable for this being the most important and successful meeting that this association has ever held.

The Local Committee of Arrangements has selected the New Willard as "Headquarters Hotel" and necessary accommodations for the meetings of the general sessions and sections, as well as the "all-day clinic" on the last day, are to be held in the commodious ball roo n on the eleventh floor of this hotel. This provides the ideal arrangements for a successful meeting, that is, all under one roof.

The reorganization proposition has been receiving most liberal support from the State Societies, which have met since the Cleveland meeting when a constitution, along the lines of the American Medical Association was tentatively adopted. This question will come up at this meeting for final action and every one interested in the perfecting of a representative National Dental Association should be present. You are respectfully requested to remember this meeting when making your vacation arrangements, as this presents an excellent opportunity to attend the meeting of the National Dental Association and visit our National Capitol.

### NATIONAL DENTAL ASSOCIATION.

WASHINGTON, D. C.

September 10th to 13th, 1912.

OPENING SESSION.

The Association will be called to order at 10:00 A. M., September 10th, in the Convention Hall of the New Willard Hotel. (*Headquarters.*)

INVOCATION—Rt. Rev. Alfred·Harding, Washington, D.C.,

ADDRESS OF WELCOME—Hon. Cuno H. Rudolph, Washington, D. C.

RESPONSE TO ADDRESS OF WELCOME—Frank O. Hetrick, Ottawa, Kansas.

PRESIDENT'S ADDRESS—Arthur R. Melendy, Knoxville, Tenn.

MODERN DENTISTRY IN GERMANY—Newell S. Jenkins, Dresden, Germany.

PROGRAM FOR GENERAL SESSIONS AND SECTIONS.

L. F. Kebler (M.D.), Washington, D. C., "Oral Dental Preparations"; F. E. Stewart (M.D.), Philadelphia, Pa., "Standardization of Materia Medica"; M. C. Smith, Lynn, Mass., "What the Government is doing to prevent Diseases"; William O. Hullick, Cincinnati, O., "Crown and Bridge Work"; J. V. Conzett, Dubuque, Iowa, "Something about Cavity Preparation for the Gold Inlay"; Leon S. Medalia (M.D.), Boston, Mass., "Pyorrhea Alveolaris, its Causes and Treatment with Vaccine"; George B. Harris, Detroit, Mich., "Pyorrhea, its Treatment by Bacterial Vaccines and Results of Animal Experimentation"; C. M. McCauley, Abilene, Texas, "The Great Need of Improvement in Quality of Commercial Alloys"; B. Holly Smith, Baltimore, Md. "Aristocracy in Dentistry—Do we need it?"; W. A. Lovett, Birmingham, Ala., "In Hoc Signo Vinces"; H. H. Johnson, Macon, Ga., "Other Methods of Making Gold Inlays"; W. R. Clack, Mason City, Iowa, "Extension for Prevention *vs.* Pin Head Cavities"; Joseph Head, Philadelphia, Pa., "Intimidation of Dentists and Dental Societies"; J. J. Moffitt, Harrisburg, Pa., "The Treatment and Filling of Root Canals"; M. L. Rhein, New York, City, "Oral Sepsis and a Consideration of its Systemic Effects"; J. F. Biddle, Allegheny, Pa., "Diagnosis and Treatment of the Important Destructive Diseases of the Dental Pulp"; Thomas B. Hartzell, Minneapolis, Minn., "Post-Operative Treatment of Pyorrhea Alveolaris. (Illustrated by Setreopticon)"; Harvey W. Wiley (M.D.), Washington, D. C., "A Consideration of the Effects that Impure Foods and Adulterated Drugs have upon the Human System"; George E. Hunt, Indianapolis, Ind., "Teeth and Health".

(The last two subjects to be given as lectures at the Wednesday evening session.)

A preliminary program was mailed to our members, essayests and clinicians August 3rd. In addition to above Literary Program, this contained 270 clinics and a number of additional ones will be incorporated in the Official Program.

RAILROAD RATES.

The Trunk Line and New England Passenger Associations have authorized a rate of a fare and three-fifths (3-5) within their territory, on certificate plan; 100 certificates required and a fee of 25 cents for validating same. The Southeastern Passenger Association advertises round-trip rates from specific points in their territory. Summer Tourists' rates to Eastern points may be secured in Western Passenger Association territory. The Central Passenger Association refused to make any concession, but Summer Tourists' rates may apply from points in this territory.

Any local railroad agent can give jurisdiction of above Associations, as well as give full particulars regarding rates and stop-over privileges from their particular point. Inquiry should be made regarding excursion rates to points east of Washington, as such transportation, with stop-over privileges, may be secured to a better advantage.

The proposed plan of reorganization is not to become effective until 1913, but our present Constitution provides that any member of a State Dental Society, or affiliated Society, is eligible to the National. The membership fee is $5.00, which pays one years' dues and includes copy of Official Transactions. Application blanks may be secured from the State Society Secretaries and must be signed by the President and Secretary and can be mailed in advance, with fee, to H. B. McFadden, Treasurer, 3505 Hamilton St., Philadelphia, Pa., or handed to him or the credential committee at time of meeting. All reputable practitioners of dentistry are invited to attend this meeting and become actively affiliated with our Association.

HOMER C. BROWN, *Recording Secretary*,
185 East State St., Columbus, O.

